# Une étude rétrospective sur l'incidence de l'insuffisance rénale chronique dans le service de Médecine Interne et Néphrologie du Centre Hospitalier Universitaire d'Antananarivo

**DOI:** 10.11604/pamj.2016.23.141.8874

**Published:** 2016-03-28

**Authors:** Benja Ramilitiana, Eliane Mikkelsen Ranivoharisoa, Mihary Dodo, Evanirina Razafimandimby, Willy Franck Randriamarotia

**Affiliations:** 1 Unité de Médecine Interne et Néphrologie, Hôpital Joseph Raseta Befelatanana, Antananarivo,Madagascar

**Keywords:** Antananarivo, hypertension, Insuffisance rénale chronique, mortalité, Antananarivo, hypertension, chronic renal failure, mortality

## Abstract

L'insuffisance rénale chronique est un problème de santé publique au niveau mondial. Dans les pays développés, cette affection survient essentiellement chez les sujets âgés, mais en Afrique, elle s'installe plutôt chez les sujets jeunes actifs. C'est une affection de lourde prise en charge dans un pays à faible revenu à cause de ses coûts. Notre but est de décrire les aspects épidémiologiques des nouveaux cas d'insuffisance rénale chronique à Madagascar. Il s'agit d'une étude rétrospective descriptive de 3 ans partant du 1er janvier 2007 au 31 décembre 2009 dans le service de Médecine Interne et Néphrologie du Centre Hospitalier Universitaire d'Antananarivo portant sur 239 patients diagnostiqués comme une insuffisance rénale chronique. L'incidence était de 8,51% parmi les patients hospitalisés dans le service. L’âge moyen des patients était de 45,4 ans avec des extrêmes de 16 et 82 ans et un sex-ratio de 1,46. Le principal antécédent était l'hypertension artérielle (59,8%). L'insuffisance rénale chronique était terminale dans 75,31% des cas (n=180). Les causes de l'insuffisance rénale chronique étaient dominées par la glomérulonéphrite chronique (40,1%), la néphroangiosclérose (35,5%). L'hémodialyse était réalisée chez 3 patients (1,26%), aucun patient n’était pas programmé pour une greffe rénale. Le taux de mortalité dans le service était de 28,87%. L'insuffisance rénale chronique est une maladie de pronostic redoutable et handicapante qui affecte les sujets jeunes à Madagascar. Son traitement reste inaccessible dans la majorité des patients. L'accent doit donc être mis principalement sur la prévention notamment une bonne prise en charge précoce des infections, de l'hypertension artérielle et du diabète pour réduire ses impacts négatifs sur la santé communautaire et publique. Le projet de la transplantation rénale - donneur vivant, traitement efficace et moins coûteux par rapport à l'hémodialyse pourrait être aussi une bonne solution chez ces sujets jeunes malgaches.

## Abstract (en)

Chronic renal failure is a global public health problem. In developed countries, this disease occurs mainly in the elderly, but in Africa it rather affects active young subjects. This disease need for expensive treatments in a low income country, because of its costs. Our aim is to describe the epidemiology of new cases of chronic renal failure in Madagascar. This is a retrospective, descriptive study of 239 patients with chronic renal failure over a 3 year period, starting from 1 January 2007 to 31 December 2009, in the Department of Internal Medicine and Nephrology at University Hospital of Antananarivo. The incidence was 8.51% among patients hospitalized in the Department. The average age of patients was 45.4 years with extremes of 16 and 82 years and a sex ratio 1,46. The main antecedent was arterial hypertension (59.8%). Chronic renal failure was terminal in 75.31% of the cases (n=180). The causes of chronic renal failure were dominated by chronic glomerulonephritis (40.1%), nephroangiosclerosis (35.5%). Hemodialysis was performed in 3 patients (1.26%), no patient was scheduled for a renal transplantation. Mortality rate in the Department was 28.87%. Chronic renal failure is a debilitating disease with a dreadful prognosis which affects young patients in Madagascar. Its treatment remains inaccessible to the majority of patients. The focus must be mainly on prevention, especially on early effective management of infections, arterial hypertension and diabetes to reduce its negative impacts on the community and public health. The project on renal transplantation: living donor, effective and less expensive treatment compared to hemodialysis could also be a good solution for these Malagasy young subjects.

## Introduction

L'insuffisance rénale chronique est un problème de santé publique au niveau mondial. En 2015, plus de 353 millions de personnes soit 5% de la population mondiale souffrent d'une insuffisance rénale chronique [1]. La prévalence varie d'un pays à un autre et l´accès aux traitements dépend du niveau socio-économique du pays concerné. Aux Etats-Unis, la prévalence estimée de tous les stades de la maladie rénale chronique est voisine de 13 p. 100 et concerne près de 20 millions d'américains, le nombre de patients en dialyse devrait y être de 650 000 en 2010 [2]. Contrairement à ce qui passe dans certains pays en voie de développement à faible revenu où l´inaccessibilité aux traitements de suppléance reste toujours la grande difficulté rencontrée. En Afrique, sa prévalence exacte n'est pas mieux documentée que dans quelques pays. En côte d´Ivoire, elle est de 5,8% des patients admis à l´Hôpital dont 5% des patients seulement ont l´accès à un traitement de suppléance [3]. Ce travail est une étude effectuée à Madagascar, dans le Centre Hospitalier Universitaire d'Antananarivo de Befelatanana dont le but est de cerner le profil épidémiologique des insuffisances rénales chroniques.

## Méthodes

Il s'agit d'une étude rétrospective, descriptive de 3 ans, réalisée dans le service de Médecine Interne et Néphrologie du Centre Hospitalo-Universitaire de Befelatanana (Antananarivo), et qui s’étend du 1^er^ janvier 2007 au 31 décembre 2009. Ont été inclus dans cette étude tous patients insuffisants rénaux chroniques de plus de quinze ans. Nous avons utilisé la définition de l'insuffisance rénale chronique selon la définition de Kidney Disease Improving Global Outcomes (KDIGO) dont la présence d'une anomalie rénale persistante au delà de trois mois que ce soit morphologique, histologique ou biologique tels la présence de marqueurs d'atteinte rénale (albuminurie, protéinurie…) associée ou non à une baisse du Débit de Filtration Glomérulaire (DFG) [4]. Les dossiers inclus étaient ceux présentant une baisse du DFG inférieure à 60 mL/mn/1,73m^2^), calculée selon la formule de Chronic Kidney Disease Epidemiology (CKD-EPI). Nous avons exclu les dossiers ayant une insuffisance rénale aiguë associée ou ceux dont les dossiers médicaux étaient inexploitables. Comme paramètres, nous avons retenu le genre, âge, facteurs de risque cardiovasculaire, le DFG, la protéinurie de 24 heures, la néphropathie causale, le traitement de l’épuration extra-rénale et la modalité de sortie. Les facteurs de risque cardiovasculaire recherchés étaient: hypertension artérielle, tabagisme, âge, diabète sucré, hypertrophie ventriculaire gauche, dyslipidémie, ménopause, obésité (indice de masse corporelle ≥5; 27Kg/m^2^), histoire d'accident vasculaire cérébral, sédentarité, antécédent familial d'accident cardiovasculaire précoce, contraception. Comme analyse statistique, nous avons utilisé le logiciel Epi-info. Nous avons défini ainsi une insuffisance rénale chronique modérée lorsque le DFG était comprise entre 30 et 59 mL/min, sévère entre 15 et 29 ml/mn et terminale lorsque le DFG était en dessous de 15 mL/mn [5]. En l'absence de biopsies rénales effectuées pour des raisons techniques, la classification étiologique de l'insuffisance rénale était fondée sur des faisceaux d'arguments cliniques et paracliniques. Ainsi, nous avons retenu: - le diagnostic de néphropathie glomérulaire chronique devant la présence d'un syndrome œdémateux ancien et/ou répété, d'une protéinurie supérieure à 3 g/24 h associée ou non à une hématurie - la néphropathie vasculaire chronique type néphroangiosclérose se base sur l'existence d'une hypertension artérielle ancienne associée à une protéinurie < 2 g/24 h, des signes de rétinopathie hypertensive et/ou une hypertrophie ventriculaire gauche, des autres complications de l'hypertension artérielle - la néphropathie diabétique se confirme devant un diabète connu plus de cinq ans, l'existence d'une protéinurie positive associée à des signes de rétinopathies diabétiques au fond d’œil, une complication dégénérative du diabète - la néphropathie tubulo-interstitielle chronique se base sur la présence d'une prise d'un médicament néphrotoxique, d'une pathologie obstructive chronique, l'existence d'une protéinurie < à 2 g/24 h associée à une leucocyturie sans germe.

## Résultats

**Figure 1:**
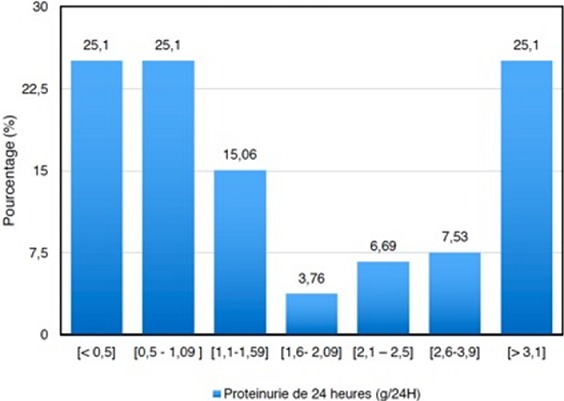
Pourcentage selon la fréquence de la protéinurie de 24 heures

Au cours de notre période d’étude, nous avons consulté 2 806 dossiers. Cinq cent soixante treize patients (20,4%) avaient un DFG inférieure à 60 mL/min/1,73m^2^), mais 239 dossiers (8,51%) avaient répondu à nos critères d'inclusion. Notre population d’étude était constituée de 142 hommes et 97 femmes avec un sex-ratio de 1,46. L’âge de nos patients variait de 16 à 82 ans avec une moyenne de 45,44 ans. La majorité des patients avaient des bas niveaux socio-économiques (65,69%), illustré par le Tableau 1. Cent soixante patients (66,95%) avaient au moins 2 facteurs de risque cardiovasculaire; l'hypertension artérielle était rencontrée chez 143 patients (59,83%), le tabagisme chez 92 patients (38,49%), l’âge chez 58 patients (24,26%), récapitulé dans le Tableau 2. L'insuffisance rénale chronique était terminale dans 75,31% des cas (n=180), sévère dans 15,06% des cas (n=36), et modérée dans 9,62% des cas (n=23). Ce niveau de dégradation de l´Insuffisance rénale est illustré par le Tableau 3. La protéinurie de 24 heures moyenne était de 3,14 g. Elle était inférieure à 0,5 g/ 24 h dans 25,1% (n=60) et supérieure à 3,1 g/ 24 h dans 25,10% des cas (n=60), les détails sont trouvés dans la Figure 1. Dans 7,11% des cas (n=17), l'insuffisance rénale chronique était de cause indéterminée. Les causes connues étaient la glomérulonéphrite chronique chez 96 patients (40,16%), la néphroangiosclérose chez 85 patients (35,56%), la néphropathie diabétique chez 30 patients (12,55%) et la néphropathie tubulo-interstitielle chronique chez 25 patients (10,46%). Parmi les 239 patients de cette cohorte, la majorité des patients (98,74%) ont eu un traitement conservateur, seulement 3 patients (soit 1,26%) avaient bénéficié d'un traitement de suppléance par hémodialyse et aucun patient n'a pu bénéficier ni la dialyse péritonéale ni un traitement de remplacement par transplantation rénale, autres types de traitements non encore disponibles. L’évolution au cours de l'hospitalisation était marquée par 69 décès, soit une mortalité hospitalière de 28,87%. En l'absence d’étude d'envergure nationale dans notre pays, les données de notre étude serviront de bases sur le profil épidémiologique de l'insuffisance rénale chronique.

**Tableau 1:** Données sur la répartition selon le niveau socio-économique

Catégorie	Nombre (n)	Pourcentage (%)
Catégorie 4: sans profession, ouvrier, femme de ménage, paysan pauvre	74	30,97
Catégorie 3: petit commerçant, cadre inférieur	83	34,72
Catégorie 2: commerçant, propriétaire de petites et moyennes entreprises, cadre niveau maîtrise	47	19,67
Catégorie 1: couche sociale aisée, riche, businessman, cadre supérieur	35	14,64
n = 239		

**Tableau 2:** Données sur la répartition selon le type de Risque Cardio-Vasculaire

Facteurs de Risque Cardio-Vasculaire	Nombre (n)	Pourcentage (%)
HTA	143	59,83
Tabac	92	38,49
Age	58	24,26
HVG	37	15,48
Dyslipidémie	32	13,38
Diabète	30	12,55
Ménopause	18	7,53
IMC≥ 27	16	6,69
AVC	14	5,85
Sédentarité	11	4,60
Antécédents familiaux de pathologie cardio-vasculaire	10	4,18
Contraception	7	2,92
n = 239		

**Tableau 3:** Données sur la répartition selon le niveau de l'insuffisance rénale chronique

Niveau de l'insuffisance rénale chronique	Nombre (n)	Pourcentage (%)
Modérée	23	9,62
Sévère	36	15,06
Terminale	180	75,31
n = 239		

## Discussion

Dans le monde, il existe une importante variation du profil épidémio-clinique de l'insuffisance rénale chronique. Certes dans les pays occidentaux, elle est plus documentée. En France, son incidence annuelle est de 80 à 90 par million d'habitants [6]. Les données africaines ne peuvent pas refléter la situation de l'insuffisance rénale chronique dans la population générale car très peu de patients ont accès aux Centres Hospitalo-Universitaires qui sont situés surtout dans les grandes villes des pays africains. En Afrique subsaharienne, sa prévalence hospitalière est de 7,5% selon une étude menée par Outtara [7]. Pour le cas de Madagascar, aucune étude antérieure a rapporté la prévalence exacte de l´insuffisance rénale chronique, d´où l´intérêt de cette étude. Au total, nous avons colligé 239 patients insuffisants rénaux chroniques. L´insuffisance rénale chronique concerne 8,51% des dossiers hospitaliers. Le sex-ratio était de 1,46 dans notre population avec une prédominance masculine, comparable à ce que Ouattara a rapporté dans ses cohortes [8]. L’âge dans notre échantillon était de 16 à 82 ans avec un âge moyen de 45,44 ans; les patients âgés de moins de 40 ans représentaient 25,52% des cas (n=61), la tranche d’âge de plus de 65 ans ne représentait que 15,48% (n= 37) des cas. Les patients insuffisants rénaux chroniques africains sont des adultes jeunes avec un âge moyen moins de cinquantaine dans d´autre étude africaine [9]. Le jeune âge des patients en Afrique est le reflet de la jeunesse de la population africaine. Ces constatations se rapprochent à celle d'une étude sur l’épidémiologie de la maladie rénale chronique réalisée au Congo; cette étude avait retrouvé un âge médian de 47 ans et avait noté une faible prévalence avant 40 ans [10]. Les résultats d'une étude réalisée en France en 2008 avaient retrouvé une incidence de l'insuffisance rénale chronique à 12,6% entre 40 et 60 ans [11]; la même étude avait révélé que cette incidence atteignait 39,4% au-delà de 60 ans. L’étude Épidémiologie de l'Insuffisance Rénale chronique dans l'Agglomération Nancéienne (EPIRAN) avait retrouvé un âge médian de 68 ans en Lorraine [12]. Nous avions noté que 66,95% de patients avaient au moins deux facteurs de risque cardiovasculaire: l'hypertension artérielle était rencontrée chez 143 patients (59,83%), le tabagisme chez 92 patients (38,49%), le facteur âge chez 58 patients (24,26%), le diabète sucré chez 30 patients (12,55%). Selon la littérature, plus il existait de facteurs de risque cardio-vasculaire, plus la fréquence de l'insuffisance rénale chronique était élevée [13]. Des travaux effectués dans plusieurs pays avaient également retrouvé l'hypertension artérielle comme étant le facteur de risque majeur associé à l'insuffisance rénale chronique [11, 12]. Une étude conduite chez des patients présentant une néphropathie chronique avait montré que le DFG déclinait deux fois plus vite chez les fumeurs que chez les non-fumeurs [14]. Lors d'une autre étude faite par Schiele F, 32% des insuffisants rénaux chroniques avec un DFG inférieur 30 mL/ min/ 1,73 m^2^), étaient des fumeurs [14]. Concernant l’âge, plusieurs auteurs avaient publié que l'insuffisance rénale chronique augmentait fortement avec l’âge; les affections de type hypertension artérielle et diabète gagnent la place dans les âges avancés dans les pays industrialisés [12]. Nous avons retrouvé une fréquence à 25,1% des cas chacune pour une protéinurie inférieure 0,5 g/ 24h et supérieure à 3,1 g/ 24h, la moyenne générale était de 3,14 g/ 24 h. Une étude réalisée à Kinshasa en 2009 avait montré une forte prévalence de la protéinurie et les principaux déterminants étaient l’âge, l'hypertension artérielle, le diabète sucré et le surpoids [10].

La recherche étiologique de l'insuffisance rénale chronique constitue une étape difficile de la prise en charge dans nos régions, la biopsie rénale est rarement réalisée ainsi que les bilans immunologiques ou des troubles auto-immuns. Cette difficulté pourrait expliquer en grande partie le taux non négligeable de causes indéterminées de l'insuffisance rénale chronique rapporté: 7,25% dans notre étude, et entre 29,2% et 62% dans des études africaines [15, 16]. Dans notre série, les causes identifiées étaient la glomérulonéphrite chronique dans 36,64% des cas, la néphroangiosclérose notée dans 32,44%, la néphrite interstitielle chronique dans 10,69% et la néphropathie diabétique dans 12,6% des cas. Dans la plupart des études africaines, les causes connues sont de loin dominées par la néphroangiosclérose avec des taux variant entre 25% et 62,1%, suivie de la néphropathie diabétique entre 11% et 20,6% [16, 17]. Dans les pays occidentaux, l'hypertension artérielle vient également en tête des causes d'insuffisance rénale chronique suivie du diabète; dans ces pays, l'espérance de vie étant prolongée pour favoriser l’émergence de ces affections dans le troisième âge [18]. Parmi les 239 patients de notre cohorte, trois patients seulement (soit 1,26%) avaient bénéficié d'un traitement de suppléance par hémodialyse. Aucun patient n'a bénéficié de dialyse péritonéale ou de transplantation rénale; ces deux techniques ne sont pas encore réalisables dans notre pays. Malgré le progrès de la médecine, la dialyse n'est pas encore de pratique courante dans les pays africains subsahariens: elle concerne seulement 5% de l´ensemble du traitement dans une étude réalisée par Diallo en Côte d'Ivoire [3]. L'insuffisance rénale chronique est un fardeau en Afrique subsaharienne avec une mortalité hospitalière de 27,8% en Côte d'Ivoire [3] et 28,87% dans notre étude; ce taux peut parfois atteindre 50% selon certains auteurs africains [15]. Cette forte mortalité est probablement en rapport avec l'inaccessibilité à la dialyse pour le plus grand nombre de patients. Concernant ces traitements, la prise en charge précoce de la maladie rénale est primordiale. L’évolution vers le stade terminal nécessitant un traitement de suppléance qui peut être potentiellement ralentie. Selon la HAS, les objectifs du traitement figurés dans le parcours de soins de la MRC consistent surtout à ralentir la progression de la maladie rénale, traiter la maladie causale, prévenir le risque cardio-vasculaire, prévenir les complications de la MRC [18]. Tous accès à différentes préventions de la MRC à travers l’éducation thérapeutique devraient être envisagés au premier plan. Comme prévention primaire, il consiste à supprimer de tous facteurs de risque à un développement à une MRC comme l'HTA et le diabète avec dosage systématique annuelle de la créatininémie. Dans ce cadre, la prévention secondaire sera ainsi l'ensemble des moyens thérapeutiques comme l´utilisation des Inhibiteurs de l´Enzyme de Conversion et des Antagonistes de Récepteurs de l´Angiotensine 2 pour ralentir la progression vers le stade terminal. Comme prévention tertiaire, c´est l´ensemble des traitements optimaux de la maladie et ses complications au décours d'un traitement conservateur [19]. Secondairement, dans les pays en voie de développement comme Madagascar, la pratique de la transplantation, un traitement plus efficace, plus efficient et moins couteux reste le traitement de premier choix chez ces sujets jeunes actifs [20].

## Conclusion

L´insuffisance rénale chronique est un événement relativement fréquent à Madagascar. Il est important de dépister précocement les sujets à risque et de surveiller de façon rapprochée la créatininémie sérique. La gestion nécessite une prise en charge adaptée selon le stade de gravité et l’âge des patients. L'arrivée au stade terminal est un chemin incontournable pour les patients, dont le coût du traitement constitue une charge lourde à l’échelon individuel et à de l'Etat. A Madagascar, les sujets concernés par l'insuffisance rénale chronique sont surtout des adultes jeunes de sexe masculin, actifs économiquement. Ces patients étaient vus dans la majorité des cas au stade terminal de la maladie. Une mauvaise prise en charge de la maladie initiale ainsi qu'une référence tardive aux néphrologues constituent une progression rapide vers ce stade terminal. L'impossibilité d'accès aux soins adéquats pour le plus grand nombre de patients marque cette étude. L'accent doit être mis sur les mesures préventives visant surtout à bien gérer les problèmes initiaux liés à la prise en charge de l'hypertension artérielle et du diabète dans la population générale. Sinon, nous lançons un appel à l'Etat Malagasy de développer la transplantation rénale avec un donneur vivant, une solution potentielle pour tous ces patients si jeunes et à moindre coût par rapport à la dialyse.

### Etat des connaissance sur le sujet

- Insuffisance rénale chronique: affecte les sujets jeunes actifs dans les pays en voie de développement.
- La plupart des patients arrivent tardivement à l'hôpital avec un stade avancé de l'IRC.
- La majorité des patients n'ont pas accès au traitement de suppléance, le taux de mortalité reste élevé.

### Contribution de notre étude a la connaissance

- Prévention à tous niveaux pour éviter la présence ou limiter la progression de l'insuffisance rénale chronique.
- Lancement de l'Education thérapeutique individuelle, ou collective pour une information, éducation et changement des comportements des patients. Comme exemple, l'observance et l'adhérence thérapeutique au cours d'une hypertension artérielle sont primordiales afin d’éviter d'effectuer une dialyse à vie.
- Demande à l'Etat Malgache de développer la transplantation rénale avec un donneur vivant apparenté à Madagascar à cause de l’âge et de l'occupation quotidienne des patients concernés.
